# Racial disparities in survival outcomes by breast tumor subtype among African American women in Memphis, Tennessee

**DOI:** 10.1002/cam4.1117

**Published:** 2017-06-14

**Authors:** Gregory Vidal, Zoran Bursac, Gustavo Miranda‐Carboni, Shelley White‐Means, Athena Starlard‐Davenport

**Affiliations:** ^1^ Division of Hematology/Oncology Department of Medicine University of Tennessee Health Science Center Memphis Tennessee 38133; ^2^ The University of Tennessee West Cancer Center Germantown Tennessee; ^3^ Division of Biostatistics and Center for Population Sciences Department of Preventive Medicine University of Tennessee Health Science Center Memphis Tennessee; ^4^ Department of Clinical Pharmacy Consortium on Health Education Economic Empowerment and Research (CHEER) University of Tennessee Health Science Center Memphis Tennessee 38163; ^5^ Department of Genetics Genomics and Informatics University of Tennessee Health Science Center Memphis Tennessee 38133

**Keywords:** African American, breast cancer, breast tumor subtypes, Memphis, racial disparity, survival outcomes

## Abstract

Racial disparities in survival among African American (AA) women in the United States have been well documented. Breast cancer mortality rates among AA women is higher in Memphis, Tennessee as compared to 49 of the largest US cities. In this study, we investigated the extent to which racial/ethnic disparities in survival outcomes among Memphis women are attributed to differences in breast tumor subtype and treatment outcomes. A total of 3527 patients diagnosed with stage I–IV breast cancer between January 2002 and April 2015 at Methodist Health hospitals and West Cancer Center in Memphis, TN were included in the analysis. Kaplan–Meier survival curves were generated and Cox proportional hazards regression were used to compare survival outcomes among 1342 (38.0%) AA and 2185 (62.0%) non‐Hispanic White breast cancer patients by race and breast tumor subtype. Over a mean follow‐up time of 29.9 months, AA women displayed increased mortality risk [adjusted hazard ratio (HR), 1.65; 95% confidence interval (CI), 1.35–2.03] and were more likely to be diagnosed at advanced stages of disease. AA women with triple‐negative breast cancer (TNBC) had the highest death rate at 26.7% compared to non‐Hispanic White women at 16.5%. AA women with TNBC and luminal B/HER2‐ breast tumors had the highest risk of mortality. Regardless of race, patients who did not have surgery had over five times higher risk of dying compared to those who had surgery. These findings provide additional evidence of the breast cancer disparity gap between AA and non‐Hispanic White women and highlight the need for targeted interventions and policies to eliminate breast cancer disparities in AA populations, particularly in Memphis, TN.

## Introduction

Breast cancer survival rates have increased significantly in the United States (US) since the early 1990s 1. However, racial disparities in breast cancer survival outcomes among African American (AA) women still persists 2, 3, 4, 5, 6. Historically, AA women have been more likely to die from breast cancer, be diagnosed with advanced stage of breast cancer, and have an increased risk of breast cancer recurrence when compared to non‐Hispanic White women 7, 8. Racial disparities in breast cancer incidence by geographic region in the US have also been documented. For instance, between 2008 and 2012, the incidence of breast cancer among AA women who reside in southern regions of the US was significantly higher than their non‐Hispanic White counterparts 8. More recently, it was reported that the incidence of breast cancer among AA women is almost equal to that of non‐Hispanic White women 8. The racial inequality in breast cancer survival has been attributed to several factors, such as socioeconomic status 9, 10, 11, 12, 13, geographical barriers to breast care 14, 15, 16, and treatment 1. Furthermore, AA women are more likely to be diagnosed with aggressive basal‐like breast tumors that are hormone receptor‐negative and that are associated with poorer breast cancer survival compared to other ethnic groups. This suggests that differences in tumor biology may also contribute to breast cancer disparities 17, 18, 19, 20, 21, 22, 23.

Recently, breast tumors have been reclassified into intrinsic subtypes based on the tumor's molecular characteristics and response to treatment 24, 25. The breast tumor subtypes include luminal A, luminal B, human epidermal growth factor receptor 2 (HER2)‐enriched [HER2‐E], and basal‐like 26, 27. Luminal and HER2 breast tumor subtypes were established based on comprehensive gene expression profiling 28. Most breast tumors diagnosed are luminal 29, 30, 31. Luminal A tumors tend to be low grade and highly express estrogen receptor (ER) and progesterone receptor (PR), but not HER2 receptors. Luminal A breast tumors respond favorably to hormonal therapies that target ER and PR 29, 30, 31. Luminal B breast tumors tend to express ER and/or PR, may express HER2, and display a high Ki‐67, a marker of cancer cell division. Women with luminal B tumors are often diagnosed at a younger age and have poorer outcomes than women with luminal A tumors 29, 30, 31, 32, 33. HER2‐E breast tumors are defined as those breast tumors that are ER negative, PR negative, and HER2 positive 34, 35. Only 5–15% of breast tumors diagnosed are HER2‐E 29. Treatments that specifically target HER2, such as Trastuzumab, also referred to as Herceptin, are very effective for HER2‐positive tumors, including HER2‐E breast tumors 36, 37, 38. Basal‐like breast tumors were first characterized by pathologists as invasive ductal carcinomas with high histological grade and mitotic rate 39, 40, 41. Most basal‐like breast tumors lack expression of ER, PR, and HER2 and are referred to as triple‐negative breast cancer (TNBC) 42. TNBC is highly metastatic and currently there are no molecular‐based targeted therapies to treat the disease 43, 44, 45.

Considering recent data highlighting a significant racial disparity gap in breast cancer survival outcomes among AA women in Memphis, TN 46, 47, this study was initiated to determine whether racial differences in survival outcomes exists between AA and non‐Hispanic White women and if these differences varied by breast tumor subtype and response to treatment.

## Materials and Methods

### Study population and design

A retrospective cohort of women who were diagnosed with breast cancer and/or treated for breast cancer at West Cancer Center or Methodist Health hospitals in Shelby County, TN was investigated. De‐identified data were provided by the Methodist Health/West Cancer Center Cancer Registry (Memphis, TN). AA and non‐Hispanic White patients who were 18 years of age or older and diagnosed with a histologically confirmed breast cancer between January 2002 and April 2015 were included in the analysis. Inclusion criteria for the analyses consisted of complete data on hormone receptor status and race. Based on the inclusion criteria, the final analysis consisted of 3527 patients, of which 1342 were AA and 2185 were non‐Hispanic White with stage 0, I, II, III, or IV breast cancer. Breast cancer stage at diagnosis used the TNM staging system as described by the American Joint Committee on Cancer Staging Manual, 7th ed 48. Patient variables recorded included race/ethnicity, age at diagnosis, tumor characteristics, stage at diagnosis, and treatment details including receipt or nonreceipt of chemotherapy and/or radiotherapy, hormonal therapy, immunotherapy (trastuzumab for HER2+ breast tumors) and surgery. The University of Tennessee Health Science Center (UTHSC) Institutional Review Board (IRB) approved the study as defined in 45 CFR 46.102(f) prior to commencement of this retrospective study.

### Exposure and all‐cause mortality and time to death

#### Race/ethnicity

Race information was self‐reported based on data derived from forms completed during the patient's clinic visit. Only women who self‐reported as being AA or non‐Hispanic White were included in the analysis. If ethnicity was unknown, women were not included in the analysis. A total of seven women were excluded because ethnicity was not known.

#### Breast tumor subtype and tumor characteristics

Information on stage, ER/PR, and HER2 hormone receptor status and tumor grade was provided by the Methodist Health/West Cancer Center Cancer Registry. Table 1 provides a definition of the breast tumor subtypes used for analysis. As previously described by Gunter von Minckwitz et al. 49, we used tumor grade to differentiate between luminal A and luminal B‐like tumors and HER2 receptor status to differentiate between luminal B/HER2‐positive and luminal B/HER2‐negative‐like breast tumors since Ki‐67 status was not available for analysis.

**Table 1:** Definition of breast tumor subtypes

Subtype	ER/PR Status	HER2 Status	Tumor Grade
Luminal A‐like	ER and/or PR+	HER2−	I and II
Luminal B/HER2+	ER and/or PR+	HER2+	Any
Luminal B/HER2−	ER and/or PR+	HER2−	III
HER2‐E	ER−/PR−	HER2+	Any
TNBC	ER−/PR−	HER2−	Any

Adapted from 60.

ER, estrogen receptor; PR: progesterone receptor; HER2: human epidermal growth factor receptor 2.

#### Covariates

Patient demographic and clinical characteristics obtained from the tumor registry files included race, age at breast cancer diagnosis, date of diagnosis, date of chemotherapy, date of radiation, date of immunotherapy, date of surgery, and date of last contact or death. The date of death was confirmed via the Social Security Death Index. Only patients with known stage, hormone receptor status, and race/ethnicity reported were included. Race/ethnicity, age (as a continuous variable), and tumor stage (as a categorical variable) were included as covariates.

### Statistical analysis

All data analyses were performed with SAS/STATv14.1 (SAS Institute Inc., Cary, NC). Descriptive statistics, including means and standard deviations, and proportions were generated for the continuous and categorical variables, for the overall sample and by race, respectively. Equality of means between the race groups were tested with the two‐sample *t*‐test, and the equality of the proportions with the chi‐squared test. Stratified Cochran–Mantel–Haenszel (CMH) analysis was applied to test the equality and estimate relative risk of death rates between race groups, by tumor subtype. Interaction between subtype and race was examined using the Breslow‐Day (BD) test. Kaplan–Meier product‐limit race generated survival curves, and by tumor subtype, and the equality of survivor function between those groups compared using the log‐rank test. Associations between survival risk and covariates of interest were tested using univariate and multivariate Cox proportional hazards regression models. Hazard ratios (HRs) and 95% confidence intervals (CIs) were calculated for each covariate to estimate the magnitude of the risk. Two‐way interactions between race and the other covariates were evaluated. Finally, we applied adjusted Cox models to estimate the HRs for race, stratified by tumor subtype. All *P*‐values were two‐sided and considered significant at the alpha 0.05 level.

## Results

### Characteristics of the studied population

Table 2 provides a detailed overview of the patient demographic and clinical characteristics, overall and by race. The cohort included a total of 3527 breast cancer patients, 1342 (38.0%) AA, and 2185 (62.0%) non‐Hispanic White. Average age at diagnosis was 56.7 years among AAs compared to 60.6 years among non‐Hispanic White women. The overall median length of follow‐up was 29.9 months. Most AA and non‐Hispanic White patients had stage I and II breast cancer. Non‐Hispanic White women were more likely to be diagnosed at stage I than AA women (57.1% vs. 40.6%; *P* < 0.0001). Fifteen percent of AA breast tumors were stage III at diagnosis compared to 9.1% among non‐Hispanic White (*P* < 0.0001). The percentage of women with stage IV breast cancer was also higher among AA women at 8.1% compared to 3.8% for non‐Hispanic White women (*P* < 0.0001). A significantly higher proportion of AA women also had triple‐negative breast cancer (27.9%) compared to non‐Hispanic White (12.8%)(*P* < 0.0001). Compared to AA women, non‐Hispanic White women were more likely to have ER‐positive breast tumors (81.7% vs. 62.5%). Most non‐Hispanic White women underwent surgery for breast cancer (94.3%), received hormonal therapy (64.7%), radiation therapy (48.8%), and chemotherapy (42.1%). Most AA women also underwent surgery for breast cancer (89.4%), but differ in the rates of chemotherapy (62.1%), radiation therapy (55.9%), and hormonal therapy (44.3%).

**Table 2:** Patient demographic and clinical characteristics

	Overall (*N* = 3527)	White (*N* = 2185)	AA (*N* = 1342)	*P* ‐value
Age at diagnosis, years (mean, SD)	59.1 (12.8)	60.6 (12.9)	56.7 (12.4)	**<0.0001**
Length of follow‐up, months (median, SD)	29.9 (18.9)	30.3 (18.4)	28.9 (19.7)	0.242
AJCC Stage %				**<0.0001**
0	0.7	0.4	1.2	
I	50.9	57.1	40.6	
II	31.6	29.6	34.9	
III	11.5	9.1	15.4	
IV	5.4	3.8	8.1	
ER positive, %	74.4	81.7	62.5	**<0.0001**
PR positive, %	65.7	72.7	54.3	**<0.0001**
HER2 overexpressed, %	16.7	15.2	19.2	**0.0022**
Molecular subtype, %				**<0.0001**
Luminal A‐like	52.4	60.7	38.8	
Luminal B/HER2+	11.3	10.8	12.1	
Luminal B/HER2‐	12.3	11.2	14.1	
HER2‐E	5.5	4.5	7.2	
TNBC	18.5	12.8	27.9	
Chemotherapy	49.3	42.1	61.2	**<0.0001**
Radiation	51.6	48.8	55.9	**<0.0001**
Hormonal therapy^a^	56.9	64.7	44.3	**<0.0001**
Immunotherapy	8.6	8.1	9.5	**0.028**
Surgery	92.5	94.3	89.4	**<0.0001**
All‐cause mortality rate	11.9	8.8	16.9	**<0.0001**

*P* < 0.05 is significant and in bold. AJCC, American Joint Committee on Cancer; ER, estrogen receptor; PR, progesterone receptor; HER2‐E, human epidermal growth factor receptor 2‐enriched; SD, standard deviation.

a Women with ER+ tumors only.

### Ethnic disparity in survival outcomes, breast tumor subtype and treatment response

Table 3 shows the percent death rate according to breast tumor subtype among AA and non‐Hispanic White women. All‐cause mortality rate was significantly higher among AA women compared to non‐Hispanic White women for luminal A‐like, luminal B/HER2‐, and triple‐negative breast tumors. AA women with TNBC had a significantly higher mortality rate at 26.7% compared to 16.5% (RR = 1.62; 95% CI, 1.18–2.22; *P* = 0.0023). Those with luminal A‐like breast tumors had the highest relative risk of mortality (RR, 1.71; 95% CI, 1.24–2.36; *P *=* *0.001) followed by AA women with luminal B/HER2‐ (RR, 1.64; 95% CI, 1.03–2.59; *P *=* *0.0339), and TNBC (RR, 1.62; 95% CI, 1.18–2.22; *P* = 0.0023), compared to their non‐Hispanic White counterparts, respectively.

**Table 3:** Percent death rate among AA and non‐Hispanic Whites according to tumor subtype

Subtype	Overall	Death rate %		RR	95% CI	*P* ‐value
		AA	White			
Luminal A‐like	7.6	10.9	6.4	1.71	1.24–2.36	**0.001**
Luminal B/HER2+	10.1	12.1	8.8	1.36	0.75–2.47	0.3038
Luminal B/HER2−	14.8	18.9	11.6	1.64	1.03–2.59	**0.0339**
HER2‐E	15.3	16.5	14.1	1.17	0.59–2.31	0.6584
TNBC	22.3	26.7	16.5	1.62	1.18–2.22	**0.0023**

*P* < 0.05 is significant and in bold.

No interaction; BD test *P* = 0.8619.

Table 4 shows models of HRs for associations between all‐cause mortality and covariates of interest in forward fashion, starting with simple univariate model that contains race only, and sequentially adjusting by breast tumor subtype, followed by age at diagnosis, then type of treatment. In all models, AA women had a statistically significant increased risk of death compared to non‐Hispanic White women. In the fully adjusted model (model 4), AA women had a 65% increased risk of death compared to non‐Hispanic White women. Regardless of race, when compared to women with luminal A‐like breast tumors, patients with TNBC and luminal B/HER2‐ breast tumors (both *P* < 0.0001) had over a two‐fold higher risk of death. We also observed that for every 5 years increase in the age at diagnosis (the older they are when diagnosed), risk of death increased by over 26% and patients who did not have appropriate procedures (radiation, hormone therapy or surgery) had higher risk of death, especially surgery with over five times increase in risk (*P* < 0.0001).

**Table 4:** Sequential models showing associations between all‐cause mortality and covariates in forward fashion

	Model 1			Model 2			Model 3			Model 4		
	HR	95% CI	*P*	HR	95% CI	*P*	HR	95% CI	*P*	HR	95% CI	*P*
Race (ref: White)												
AA	1.94	1.61–2.36	**<0.0001**	1.59	1.31–1.95	**<0.0001**	1.89	1.65–2.15	**<0.0001**	1.65	1.42–1.86	**<0.0001**
Subtype (ref: Luminal A‐like)												
Luminal B/HER2+				1.21	0.84–1.72	0.3077	1.39	0.97–1.99	**0.0704**	1.01	0.69–1.46	0.9726
Luminal B/HER2−				1.94	1.44–2.61	**<0.0001**	2.26	1.67–3.04	**<0.0001**	2.14	1.58–2.91	**<0.0001**
HER2‐E				1.91	1.26–2.86	**0.002**	2.55	1.69–3.84	**<0.0001**	1.16	0.74–1.81	0.5171
TNBC				2.83	2.22–3.59	**<0.0001**	3.55	2.79–4.53	**<0.0001**	2.49	1.85–3.36	**<0.0001**
Age at diagnosis (5 years)							1.26	1.21–1.31	**<0.0001**	1.19	1.14–1.23	**<0.0001**
No chemotherapy										0.79	0.62–1.01	**0.0642**
No radiation										1.91	1.53–2.41	**<0.0001**
No hormone therapy										1.58	1.23–2.03	**<0.0001**
No surgery										5.66	4.43–7.25	**<0.0001**

Since AA TNBC patients had the worst survival outcomes, a multivariate Cox‐regression analysis was performed to determine the risk between race/ethnicity and all‐cause mortality stratified by breast tumor subtype (Table 5). A statistically significant increase in risk of death for luminal A‐like tumors (adjusted HR, 1.87; 95% CI, 1.33–2.62; *P* = 0.0003), luminal B/HER2‐ tumors (adjusted HR, 2.07; 95% CI, 1.24–3.44; *P* = 0.052), and TNBC (adjusted HR, 1.92; 95% CI, 1.34–2.75; *P* = 0.0008) was observed for AA women compared to their non‐Hispanic White counterparts. These differences were found both in the age‐adjusted and age adjusted‐/procedure‐adjusted models. We did not observe significant differences in risk of luminal B/HER2 +  and HER2 type breast tumor subtypes between AA and non‐Hispanic White women.

**Table 5:** HRs for all‐cause‐mortality by race/ethnicity for each breast tumor subtype

Subtype race	Model 1^a^			Model 2^b^		
	HR	95% CI	*P*	HR	95% CI	*P*
Luminal A‐like						
White	1.00			1.00		
AA	1.87	1.33–2.62	**0.0003**	1.43	1.01–2.09	**0.0478**
Luminal B/HER2+						
White	1.00			1.00		
AA	1.44	0.76–2.74	0.265	1.15	0.57–2.31	0.6996
Luminal B/HER2‐						
White	1.00			1.00		
AA	2.07	1.24–3.44	**0.052**	1.97	1.17–3.32	**0.0103**
HER2‐E						
White	1.00			1.00		
AA	2.13	0.92–4.97	0.0787	1.58	0.66–3.82	0.3065
TNBC						
White	1.00			1.00		
AA	1.92	1.34–2.75	**0.0004**	1.87	1.29–2.69	**0.0008**

a Age‐adjusted.

b Adjusted for age and whether or not received surgery, chemotherapy, radiation, and/or hormone therapy.

Bold values represent *p*‐values that are statistically significant. *P* < 0.05 is significant.

### Ethnic differences in survival outcomes by race/ethnicity and breast tumor subtypes

**Figure 1:**
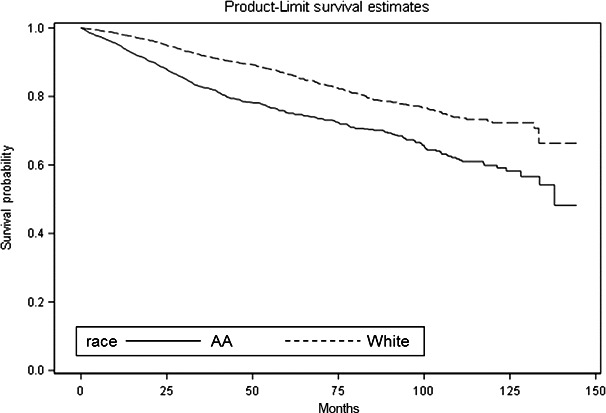
Overall survival (OS) days after diagnosis, by race.

**Figure 2:**
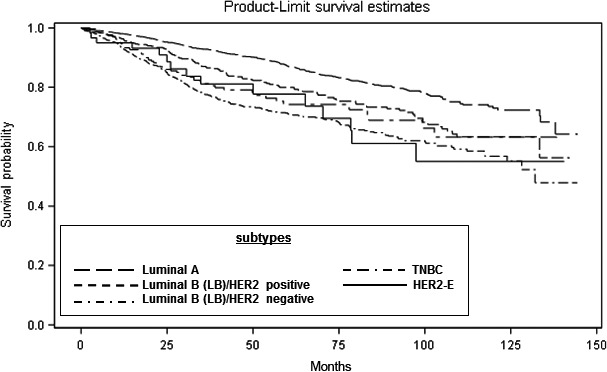
Overall survival (OS) days after diagnosis, stratified by breast tumor subtype.

We examined differences in disparity of survival functions by race/ethnicity (Fig. 1) and breast tumor subtype (Fig. 2). Figure 1 illustrates the Kaplan–Meier survival curves with the corresponding log‐rank *P*‐values for the overall survival by race/ethnicity. Overall, AA breast cancer patients had significantly shorter median survival (time to death) compared to non‐Hispanic White patients (*P* < 0.0001). We also observed a significant racial/ethnic difference in survival during the first 5 years (log‐rank *P* < 0.0001), but not during the second consecutive 5 years (log‐rank *P* = 0.1499). When examining survival outcomes by breast tumor subtypes regardless of race/ethnicity, patients with HER2‐E and TNBC had significantly shorter median survival time (*P* < 0.0001) compared to patients with luminal A and luminal B‐like tumors (Fig. 2).

## Discussion

In this study, we demonstrate that AA breast cancer patients have poorer survival outcomes than non‐Hispanic White women in Memphis, TN. It is well documented that the burden of breast cancer is greatest among women of African descent compared to other racial/ethnic populations 2, 7, 50. Newman et al. showed in a meta‐analysis of 20 studies consisting of 14,103 AA and 76,111 non‐Hispanic White women that being an AA woman is a significant and independent predictor of poor breast cancer outcomes, even after accounting for socioeconomic status 3. Published data by Whitman et al. also provided strong evidence of increased race‐specific breast cancer mortality rates among AA women in 25 of the largest US cities 47. In that study, Whitman et al. reported that between years 2005 and 2007 age‐adjusted breast cancer mortality rates among AA women compared to non‐Hispanic White women was highest in Memphis, TN compared to 24 of the largest US cities 47. Two years later, Hunt and Whitman found that the breast cancer mortality disparity gap had not changed but instead was steadily increasing and Memphis still remained the top US city for breast cancer deaths among AA women 46.

While the cause of breast cancer disparities seen in AA populations is multifaceted encompassing factors such as age, stage of breast cancer, poverty, and socioeconomic status 3, 51, 52, other factors including differences in tumor biology are highly likely to influence breast cancer survival outcomes 22, 53, 54, 55. For instance, it is well documented that AA women are more likely to present with early onset breast tumors that are triple‐negative and non‐responsive to hormone receptor therapies 22, 56, 57. In our study, we found that AA women with TNBC had a significantly higher relative risk of dying compared to non‐Hispanic White women. Regardless of race, when compared to women with luminal A‐like breast tumors, those with TNBC had a significantly higher risk of death (*P* < 0.0001). We acknowledge that the incidence of TNBC among AA women in our study was slightly lower, but not significant, than the national average. This difference could be related to discrepancies in referral patterns based on ethnicity 58, 59 and breast cancer subtype in the city of Memphis. Interestingly, we also observed that AA women with luminal B/HER2‐ breast tumors had a significantly higher relative risk of mortality than non‐Hispanic White women. Our findings are consistent with those of Warner et al., who recently reported that AA were 56% more likely to die as a result of luminal B‐like tumors 60. However, contrary to our findings and others 53, 61, Warner et al. did not observe any racial disparities in survival outcomes for TNBC patients. This could be attributed to differences in geographical location as both our study and those of Lund et al. captured patients in southern regions of the US, specifically Memphis, TN and Atlanta, Georgia. In addition, our study included patients diagnosed from 2002 to 2015 compared to those of Warner et al. who included patients diagnosed from 2000 to 2007. Therefore, their study may not reflect the increasing incidence of TNBC seen among AA populations during later years. In addition, there are unique differences in treatment of breast cancer by breast tumor subtype in more modern times. There is now an increased reliance on neoadjuvant chemotherapeutic treatment approaches, which have altered the surgical and radiation approaches as well as the increased reliance on non‐anthracycline‐based chemotherapies 49, 50. The influence of these factors on outcomes was not assessed in the study.

Another important finding in our study was the underuse of hormone therapy among AA and non‐Hispanic White breast cancer patients with luminal breast tumors. We found that 82.7% of non‐Hispanic White women and 65% of AA patients had luminal breast tumors, yet only 64.7% and 44.3% received hormone therapy, respectively. The reason for the underuse of hormone therapy in our patient population is unclear; however, there is established evidence that disparities in care, as well as increase rates of nonadherence is more prevalent in the AA population. Lack of insurance, poor access to care, lack of trust in the system, and increased toxicity—all influence adherence and are factors that can influence treatment. Furthermore, Warner et al. found the greatest disparity in survival was in women with luminal subtypes 60.

Similar to Warner et al., we also observed significant disparities among AA women with luminal A‐like and luminal B/HER2‐ breast tumors. Why AA women diagnosed with luminal A‐like breast tumors are dying is a major concern as these tumors are generally the least aggressive of all breast tumor types, are identified at early stages of disease, and are treated using targeted therapies. This finding highlights the magnitude of racial disparities with regard to breast cancer treatment, breast tumor subtype, and follow‐up after treatment among AA breast cancer patients. It is well documented that despite recommendations for breast cancer care, AA women are less likely to receive appropriate follow‐up care 62. This may be due to financial barriers to care 62, 63, psychosocial care to reduce fear, patient refusal of treatment, or other factors such as treatment delay 64, 65. A number of published studies have reported racial disparities in treatment delay in AA women 66, 67, 68, particularly in the South 69, 70.

A limitation of our study is that we did not have information available for specific cause of death; however, given breast cancer diagnosis for these patients, we can speculate that it was at least a contributing cause if not the leading cause of death. In addition, we were unable to distinguish between basal‐like breast tumors and TNBC because we did not have histopathological data on Ki‐67 status, a reporter of cancer cell division used for classification of TNBC into two subtypes based on response and prognosis 71. Thus, we used reported receptor status and tumor grade as surrogates for molecular subtype to estimate luminality as previously described 49, 60, 72.

Despite these limitations, our findings are consistent with those from other large epidemiological studies that report similar breast cancer mortality trends among AA breast cancer patients. Specifically, we confirm that AA women have a higher percentage and risk of mortality than non‐Hispanic White women. We believe that our data highlights the magnitude of racial disparities seen in Memphis, TN and may reflect other disparate populations. Few studies have examined the impact of breast tumor subtype on survival outcomes, particularly in AA women. Our study is the first study to identify racial disparities by breast tumor subtypes and survival between AA and non‐Hispanic White women in Memphis, TN, a city with significant breast cancer disparities in women of African descent. We hope that our work sheds new insight on the importance of reducing breast cancer disparities in minority populations and highlights the need for targeted interventions and policies aimed at reducing barriers and behaviors that influence breast cancer disparities in AA women, particularly in Memphis, TN. In summary, we found that AA women with breast cancer had poorer overall survival relative to non‐Hispanic White women and this difference was greatest among women with TNBC. Further research is necessary to understand the biological factors that contribute to the increased number of deaths due to TNBC in women of African descent.

## Conflicts of Interests

The authors do not have any conflicts of interests to disclose.

## References

[1] DeSantis, C. E. , R. L. Siegel , A. G. Sauer , K. D. Miller , S. A. Fedewa , K. I. Alcaraz , et al. 2016 Cancer statistics for African Americans, 2016: progress and opportunities in reducing racial disparities. CA Cancer J. Clin. 66:290–308. 26910411 10.3322/caac.21340

[2] Maskarinec, G. , C. Sen , K. Koga , and S. M. Conroy . 2011 Ethnic differences in breast cancer survival: status and determinants. Womens Health (Lond). 7:677–687. 22040209 10.2217/whe.11.67 PMC3256927

[3] Newman, L. A. , K. A. Griffith , I. Jatoi , M. S. Simon , J. P. Crowe , and G. A. Colditz . 2006 Meta‐analysis of survival in African American and white American patients with breast cancer: ethnicity compared with socioeconomic status. J. Clin. Oncol. 24:1342–1349. 16549828 10.1200/JCO.2005.03.3472

[4] LeMarchand, L. , L. N. Kolonel , and A. M. Nomura . 1984 Relationship of ethnicity and other prognostic factors to breast cancer survival patterns in Hawaii. J. Natl Cancer Inst. 73:1259–1265. 6595437

[5] Ooi, S. L. , M. E. Martinez , and C. I. Li . 2011 Disparities in breast cancer characteristics and outcomes by race/ethnicity. Breast Cancer Res. Treat. 127:729–738. 21076864 10.1007/s10549-010-1191-6 PMC3081535

[6] Li, C. I. , K. E. Malone , and J. R. Daling . 2003 Differences in breast cancer stage, treatment, and survival by race and ethnicity. Arch. Intern. Med. 163:49–56. 12523916 10.1001/archinte.163.1.49

[7] Danforth, D. N. Jr . 2013 Disparities in breast cancer outcomes between Caucasian and African American women: a model for describing the relationship of biological and nonbiological factors. Breast Cancer Res. 15:208. 23826992 10.1186/bcr3429 PMC3706895

[8] DeSantis, C. E. , S. A. Fedewa , A. Goding Sauer , J. L. Kramer , R. A. Smith , and A. Jemal . 2016 Breast cancer statistics, 2015: convergence of incidence rates between black and white women. CA Cancer J. Clin. 66:31–42. 26513636 10.3322/caac.21320

[9] Parise, C. A. , and V. Caggiano . 2013 Disparities in race/ethnicity and socioeconomic status: risk of mortality of breast cancer patients in the California Cancer Registry, 2000‐2010. BMC Cancer 13:449. 24083624 10.1186/1471-2407-13-449 PMC3850736

[10] Yu, X. Q. 2009 Socioeconomic disparities in breast cancer survival: relation to stage at diagnosis, treatment and race. BMC Cancer 9:364. 19828016 10.1186/1471-2407-9-364 PMC2770567

[11] Gerend, M. A. , and M. Pai . 2008 Social determinants of Black‐White disparities in breast cancer mortality: a review. Cancer Epidemiol. Biomarkers Prev. 17:2913–2923. 18990731 10.1158/1055-9965.EPI-07-0633

[12] Campbell, R. T. , X. Li , T. A. Dolecek , R. E. Barrett , K. E. Weaver , and R. B. Warnecke . 2009 Economic, racial and ethnic disparities in breast cancer in the US: towards a more comprehensive model. Health Place 15:855–864. 19307146 10.1016/j.healthplace.2009.02.007 PMC2754280

[13] Tian, N. , J. G. Wilson , and F. B. Zhan . 2011 Spatial association of racial/ethnic disparities between late‐stage diagnosis and mortality for female breast cancer: where to intervene? Int. J. Health Geogr. 10:24. 21463525 10.1186/1476-072X-10-24 PMC3079591

[14] Tatalovich, Z. , L. Zhu , A. Rolin , D. R. Lewis , L. C. Harlan , and D. M. Winn . 2015 Geographic disparities in late stage breast cancer incidence: results from eight states in the United States. Int. J. Health Geogr. 14:31. 26497363 10.1186/s12942-015-0025-5 PMC4619382

[15] Dai, D. 2010 Black residential segregation, disparities in spatial access to health care facilities, and late‐stage breast cancer diagnosis in metropolitan Detroit. Int. J. Health Geogr. 16:1038–1052. 10.1016/j.healthplace.2010.06.012 20630792

[16] Keller, D. , C. Guilfoyle , and J. Sariego . 2011 Geographical influence on racial disparity in breast cancer presentation in the United States. Am. Surg. 77:933–936. 21944362

[17] Newman, L. A. 2016 Parsing the Etiology of Breast Cancer Disparities. J. Clin. Oncol. 34:1013–1014. 26786919 10.1200/JCO.2015.65.1877

[18] Trock, B. J. 1996 Breast cancer in African American women: epidemiology and tumor biology. Breast Cancer Res. Treat. 40:11–24. 8888149 10.1007/BF01805999

[19] Amend, K. , D. Hicks , and C. B. Ambrosone . 2006 Breast cancer in African‐American women: differences in tumor biology from European‐American women. Cancer Res. 66:8327–8330. 16951137 10.1158/0008-5472.CAN-06-1927

[20] Verma, R. , R. L. Bowen , S. E. Slater , F. Mihaimeed , and J. L. Jones . 2012 Pathological and epidemiological factors associated with advanced stage at diagnosis of breast cancer. Br. Med. Bull. 103:129–145. 22864058 10.1093/bmb/lds018

[21] Boyle, P . 2012 Triple‐negative breast cancer: epidemiological considerations and recommendations. Ann. Oncol. 23(Suppl 6):vi7–vi12. 23012306 10.1093/annonc/mds187

[22] Dietze, E. C. , C. Sistrunk , G. Miranda‐Carboni , R. O'Regan , and V. L. Seewaldt . 2015 Triple‐negative breast cancer in African‐American women: disparities versus biology. Nat. Rev. Cancer 15:248–254. 25673085 10.1038/nrc3896 PMC5470637

[23] Sturtz, L. A. , J. Melley , K. Mamula , C. D. Shriver , and R. E. Ellsworth . 2014 Outcome disparities in African American women with triple negative breast cancer: a comparison of epidemiological and molecular factors between African American and Caucasian women with triple negative breast cancer. BMC Cancer 14:62. 24495414 10.1186/1471-2407-14-62 PMC3916697

[24] Polyak, K. 2011 Heterogeneity in breast cancer. J. Clin. Invest. 121:3786–3788. 21965334 10.1172/JCI60534 PMC3195489

[25] Prat, A. , O. Karginova , J. S. Parker , C. Fan , X. He , L. Bixby , et al. 2013 Characterization of cell lines derived from breast cancers and normal mammary tissues for the study of the intrinsic molecular subtypes. Breast Cancer Res. Treat. 142:237–255. 24162158 10.1007/s10549-013-2743-3 PMC3832776

[26] Sorlie, T. , C. M. Perou , R. Tibshirani , T. Aas , S. Geisler , H. Johnsen , et al. 2001 Gene expression patterns of breast carcinomas distinguish tumor subclasses with clinical implications. Proc. Natl Acad. Sci. USA 98:10869–10874. 11553815 10.1073/pnas.191367098 PMC58566

[27] Perou, C. M. , T. Sorlie , M. B. Eisen , M. van de Rijn , S. S. Jeffrey , C. A. Rees , et al. 2000 Molecular portraits of human breast tumours. Nature 406:747–752. 10963602 10.1038/35021093

[28] Schnitt, S. J. 2010 Classification and prognosis of invasive breast cancer: from morphology to molecular taxonomy. Mod. Pathol. 23(Suppl 2):S60–S64. 20436504 10.1038/modpathol.2010.33

[29] Voduc, K. D. , M. C. Cheang , S. Tyldesley , K. Gelmon , T. O. Nielsen , and H. Kennecke . 2010 Breast cancer subtypes and the risk of local and regional relapse. J. Clin. Oncol. 28:1684–1691. 20194857 10.1200/JCO.2009.24.9284

[30] Arvold, N. D. , A. G. Taghian , A. Niemierko , R. F. Abi Raad , M. Sreedhara , P. L. Nguyen , et al. 2011 Age, breast cancer subtype approximation, and local recurrence after breast‐conserving therapy. J. Clin. Oncol. 29:3885–3891. 21900114 10.1200/JCO.2011.36.1105 PMC3189090

[31] Metzger‐Filho, O. , Z. Sun , G. Viale , K. N. Price , D. Crivellari , R. D. Snyder , et al. 2013 Patterns of Recurrence and outcome according to breast cancer subtypes in lymph node‐negative disease: results from international breast cancer study group trials VIII and IX. J. Clin. Oncol. 31:3083–3090. 23897954 10.1200/JCO.2012.46.1574 PMC3753700

[32] Lund, M. J. , E. N. Butler , B. Y. Hair , K. C. Ward , J. H. Andrews , G. Oprea‐Ilies , et al. 2010 Age/race differences in HER2 testing and in incidence rates for breast cancer triple subtypes: a population‐based study and first report. Cancer 116:2549–2559. 20336785 10.1002/cncr.25016

[33] Haque, R. , S. A. Ahmed , G. Inzhakova , J. Shi , C. Avila , J. Polikoff , et al. 2012 Impact of breast cancer subtypes and treatment on survival: an analysis spanning two decades. Cancer Epidemiol. Biomarkers Prev. 21:1848–1855. 22989461 10.1158/1055-9965.EPI-12-0474 PMC3467337

[34] Bastien, R. R. , A. Rodriguez‐Lescure , M. T. Ebbert , A. Prat , B. Munarriz , L. Rowe , et al. 2012 PAM50 breast cancer subtyping by RT‐qPCR and concordance with standard clinical molecular markers. BMC Med. Genomics 5:44. 23035882 10.1186/1755-8794-5-44 PMC3487945

[35] Prat, A. , L. A. Carey , B. Adamo , M. Vidal , J. Tabernero , J. Cortes , et al. 2014 Molecular features and survival outcomes of the intrinsic subtypes within HER2‐positive breast cancer. J. Natl Cancer Inst. 106:dju152 https://doi.org/1093/jnci/dju152. 25139534 10.1093/jnci/dju152 PMC4151853

[36] Platet, N. , A. M. Cathiard , M. Gleizes , and M. Garcia . 2004 Estrogens and their receptors in breast cancer progression: a dual role in cancer proliferation and invasion. Crit. Rev. Oncol. Hematol. 51:55–67. 15207254 10.1016/j.critrevonc.2004.02.001

[37] Smith, I. E. , and M. Dowsett . 2003 Aromatase inhibitors in breast cancer. N. Engl. J. Med. 348:2431–2442. 12802030 10.1056/NEJMra023246

[38] Piccart‐Gebhart, M. J. , M. Procter , B. Leyland‐Jones , A. Goldhirsch , M. Untch , I. Smith , et al. 2005 Trastuzumab after adjuvant chemotherapy in HER2‐positive breast cancer. N. Engl. J. Med. 353:1659–1672. 16236737 10.1056/NEJMoa052306

[39] Livasy, C. A. , G. Karaca , R. Nanda , M. S. Tretiakova , O. I. Olopade , D. T. Moore , et al. 2006 Phenotypic evaluation of the basal‐like subtype of invasive breast carcinoma. Mod. Pathol. 19:264–271. 16341146 10.1038/modpathol.3800528

[40] Fulford, L. G. , D. F. Easton , J. S. Reis‐Filho , A. Sofronis , C. E. Gillett , S. R. Lakhani , et al. 2006 Specific morphological features predictive for the basal phenotype in grade 3 invasive ductal carcinoma of breast. Histopathology 49:22–34. 16842243 10.1111/j.1365-2559.2006.02453.x

[41] Fadare, O. , and F. A. Tavassoli . 2007 The phenotypic spectrum of basal‐like breast cancers: a critical appraisal. Adv. Anat. Pathol. 14:358–373. 17717437 10.1097/PAP.0b013e31814b26fe

[42] Diaz, L. K. , V. L. Cryns , W. F. Symmans , and N. Sneige . 2007 Triple negative breast carcinoma and the basal phenotype: from expression profiling to clinical practice. Adv. Anat. Pathol. 14:419–430. 18049131 10.1097/PAP.0b013e3181594733

[43] Podo, F. , L. M. Buydens , H. Degani , R. Hilhorst , E. Klipp , I. S. Gribbestad , et al. 2010 Triple‐negative breast cancer: present challenges and new perspectives. Mol. Oncol. 4:209–229. 20537966 10.1016/j.molonc.2010.04.006 PMC5527939

[44] Bhattacharya, R. , K. Banerjee , N. Mukherjee , M. Sen , and A. Mukhopadhyay . 2017 From molecular insight to therapeutic strategy: The holistic approach for treating triple negative breast cancer. Pathol. Res. Pract. 213:177–182. 28215644 10.1016/j.prp.2017.01.001

[45] Hudis, C. A. , and L. Gianni . 2011 Triple‐negative breast cancer: an unmet medical need. Oncologist 16 (Suppl 1):1–11. 10.1634/theoncologist.2011-S1-01 21278435

[46] Hunt, B. R. , S. Whitman , and M. S. Hurlbert . 2014 Increasing black: white disparities in breast cancer mortality in the 50 largest cities in the United States. Cancer Epidemiol. 38:118–123. 24602836 10.1016/j.canep.2013.09.009

[47] Whitman, S. , J. Orsi , and M. Hurlbert . 2012 The racial disparity in breast cancer mortality in the 25 largest cities in the United States. Cancer Epidemiol. 36:e147–e151. 22443886 10.1016/j.canep.2011.10.012

[48] Edge, S. B. , and C. C. Compton . 2010 The American Joint Committee on Cancer: the 7th edition of the AJCC cancer staging manual and the future of TNM. Ann. Surg. Oncol. 17:1471–1474. 20180029 10.1245/s10434-010-0985-4

[49] von Minckwitz, G. , M. Untch , J. U. Blohmer , S. D. Costa , H. Eidtmann , P. A. Fasching , et al. 2012 Definition and impact of pathologic complete response on prognosis after neoadjuvant chemotherapy in various intrinsic breast cancer subtypes. J. Clin. Oncol. 30:1796–1804. 22508812 10.1200/JCO.2011.38.8595

[50] Newman, L. A. 2014 Breast cancer disparities: high‐risk breast cancer and African ancestry. Surg. Oncol. Clin. N. Am. 23:579–592. 24882352 10.1016/j.soc.2014.03.014

[51] Leopold, C. , A. K. Wagner , F. Zhang , C. Y. Lu , C. Earle , L. Nekhlyudov , et al. 2016 Racial disparities in all‐cause mortality among younger commercially insured women with incident metastatic breast cancer. Breast Cancer Res. Treat. 158:333–340. 27342456 10.1007/s10549-016-3875-z PMC6464367

[52] Newman, L. A. , J. Mason , D. Cote , Y. Vin , K. Carolin , D. Bouwman , et al. 2002 African‐American ethnicity, socioeconomic status, and breast cancer survival: a meta‐analysis of 14 studies involving over 10,000 African‐American and 40,000 White American patients with carcinoma of the breast. Cancer 94:2844–2854. 12115371 10.1002/cncr.10575

[53] Lund, M. J. , K. F. Trivers , P. L. Porter , R. J. Coates , B. Leyland‐Jones , O. W. Brawley , et al. 2009 Race and triple negative threats to breast cancer survival: a population‐based study in Atlanta GA. Breast Cancer Res. Treat. 113:357–370. 18324472 10.1007/s10549-008-9926-3

[54] Daly, B. , and O. I. Olopade . 2015 A perfect storm: How tumor biology, genomics, and health care delivery patterns collide to create a racial survival disparity in breast cancer and proposed interventions for change. CA Cancer J. Clin. 65:221–238. 25960198 10.3322/caac.21271

[55] Brewster, A. M. , M. Chavez‐MacGregor , and P. Brown . 2014 Epidemiology, biology, and treatment of triple‐negative breast cancer in women of African ancestry. Lancet Oncol. 15:e625–e634. 25456381 10.1016/S1470-2045(14)70364-X PMC4413447

[56] Carey, L. A. , C. M. Perou , C. A. Livasy , L. G. Dressler , D. Cowan , K. Conway , et al. 2006 Race, breast cancer subtypes, and survival in the Carolina Breast Cancer Study. JAMA 295:2492–2502. 16757721 10.1001/jama.295.21.2492

[57] Parise, C. A. , K. R. Bauer , and V. Caggiano . 2010 Variation in breast cancer subtypes with age and race/ethnicity. Crit. Rev. Oncol. Hematol. 76:44–52. 19800812 10.1016/j.critrevonc.2009.09.002

[58] Cragun, D. , D. Bonner , J. Kim , M. R. Akbari , S. A. Narod , A. Gomez‐Fuego , et al. 2015 Factors associated with genetic counseling and BRCA testing in a population‐based sample of young Black women with breast cancer. Breast Cancer Res. Treat. 151:169–176. 25868867 10.1007/s10549-015-3374-7 PMC4503247

[59] Goodman, L. R. , U. Balthazar , J. Kim , and J. E. Mersereau . 2012 Trends of socioeconomic disparities in referral patterns for fertility preservation consultation. Hum. Reprod. 27:2076–2081. 22552688 10.1093/humrep/des133 PMC6457079

[60] Warner, E. T. , R. M. Tamimi , M. E. Hughes , R. A. Ottesen , Y. N. Wong , S. B. Edge , et al. 2015 Racial and ethnic differences in breast cancer survival: mediating effect of tumor characteristics and sociodemographic and treatment factors. J. Clin. Oncol. 33:2254–2261. 25964252 10.1200/JCO.2014.57.1349 PMC4486344

[61] Akinyemiju, T. , J. X. Moore , and S. F. Altekruse . 2015 Breast cancer survival in African‐American women by hormone receptor subtypes. Breast Cancer Res. Treat. 153:211–218. 26250393 10.1007/s10549-015-3528-7 PMC6043784

[62] Palmer, N. R. , K. E. Weaver , S. P. Hauser , J. A. Lawrence , J. Talton , L. D. Case , et al. 2015 Disparities in barriers to follow‐up care between African American and White breast cancer survivors. Support. Care Cancer 23:3201–3209. 25821145 10.1007/s00520-015-2706-9 PMC4586316

[63] Short, L. J. , M. D. Fisher , P. M. Wahl , M. B. Kelly , G. D. Lawless , S. White , et al. 2010 Disparities in medical care among commercially insured patients with newly diagnosed breast cancer: opportunities for intervention. Cancer 116:193–202. 19877115 10.1002/cncr.24691

[64] White‐Means, S. , M. Rice , J. Dapremont , B. Davis , and J. Martin . 2016 African American women: surviving breast cancer mortality against the highest odds. Int. J. Environ. Res. Public Health 13: https://doi.org/10.3390/ijerph13010006. 10.3390/ijerph13010006 PMC4730397 26703655

[65] Ashing‐Giwa, K. T. , G. Padilla , J. Tejero , J. Kraemer , K. Wright , A. Coscarelli , et al. 2004 Understanding the breast cancer experience of women: a qualitative study of African American, Asian American. Latina and Caucasian cancer survivors. Psychooncology 13:408–428. 15188447 10.1002/pon.750 PMC1618782

[66] Sheppard, V. B. , B. A. Oppong , R. Hampton , F. Snead , S. Horton , F. Hirpa , et al. 2015 Disparities in breast cancer surgery delay: the lingering effect of race. Ann. Surg. Oncol. 22:2902–2911. 25652051 10.1245/s10434-015-4397-3

[67] George, P. , S. Chandwani , M. Gabel , C. B. Ambrosone , G. Rhoads , E. V. Bandera , et al. 2015 Diagnosis and surgical delays in African American and white women with early‐stage breast cancer. J. Womens Health (Larchmt). 24:209–217. 25650628 10.1089/jwh.2014.4773 PMC4442576

[68] McGee, S. A. , D. D. Durham , C. K. Tse , and R. C. Millikan . 2013 Determinants of breast cancer treatment delay differ for African American and White women. Cancer Epidemiol. Biomarkers Prev. 22:1227–1238. 23825306 10.1158/1055-9965.EPI-12-1432 PMC3719384

[69] Connors, S. K. , M. S. Goodman , L. Noel , N. N. Chavakula , D. Butler , S. Kenkel , et al. 2015 Breast cancer treatment among African American women in north St. Louis, Missouri. J. Urban Health 92:67–82. 24912599 10.1007/s11524-014-9884-5 PMC4338122

[70] Johnston, E. M. , S. C. Blake , K. L. Andes , L. N. Chien , and E. K. Adams . 2014 Breast cancer treatment experiences by race and location in Georgia's Women's Health Medicaid Program. Womens Health Issues 24:e219–e229. 24560120 10.1016/j.whi.2014.01.002

[71] Keam, B. , S. A. Im , K. H. Lee , S. W. Han , D. Y. Oh , J. H. Kim , et al. 2011 Ki‐67 can be used for further classification of triple negative breast cancer into two subtypes with different response and prognosis. Breast Cancer Res. 13: R22. 21366896 10.1186/bcr2834 PMC3219180

[72] Cheang, M. C. , D. Voduc , C. Bajdik , S. Leung , S. McKinney , S. K. Chia , et al. 2008 Basal‐like breast cancer defined by five biomarkers has superior prognostic value than triple‐negative phenotype. Clin. Cancer Res. 14:1368–1376. 18316557 10.1158/1078-0432.CCR-07-1658

